# Peach Leaf Extract (*Prunus persica* L.) Mitigates Metabolic Syndrome and Oxidative Stress in High-Fructose Diet Rats

**DOI:** 10.3390/plants14091332

**Published:** 2025-04-28

**Authors:** Djihane Bali, Zoubida Mami-Soualem, Nabila Belyagoubi-Benhammou, Nassima Benzazoua, Chahrazed Belarbi, Youssouf Kachekouche, Waleed Aldahmash, Md Ataur Rahman, Abdel Halim Harrath

**Affiliations:** 1 Natural Products Laboratory, Department of Biology, Faculty of Natural and Life Sciences, Earth and Universe Sciences, University Abou-Bekr Belkaïd, Tlemcen 13000, Algeria; djihane13@live.com (D.B.); mamizoubida@hotmail.fr (Z.M.-S.); chahrazed.be_96@outlook.fr (C.B.); 2 Laboratory of Histology-Embryology, Faculty of Medicine Dr. Benzerdjeb Benaouda, University of Tlemcen, Tlemcen 13000, Algeria; benznew007@gmail.com; 3 Department of Biology, Faculty of SNV, University of Hassiba Benbouali, Chlef 02000, Algeria; youcef.kache13@gmail.com; 4 Department of Zoology, College of Science, King Saud University, Riyadh 11451, Saudi Arabia; dhmash_28@yahoo.com; 5 Department of Oncology, Karmanos Cancer Institute, School of Medicine, Wayne State University, Detroit, MI 48201, USA; rahman23@wayne.edu

**Keywords:** *Prunus persica* L., phenolic compounds, oxidative stress, metabolic syndrome, high-fructose diet

## Abstract

This study aimed to evaluate the protective effects of peach leaf extract (*Prunus persica* L.) against metabolic syndrome and oxidative stress in *Wistar* rats subjected to a high-fructose diet. The *Wistar* rats were divided into groups and fed a high-fructose diet, with or without supplementation of peach leaf extract. The extract was characterized by its bioactive compounds, including an organic acid yield of 53.8%, total phenolic content (TPC) of 273.36 ± 1.929 mg GAE/g DW, flavonoid content (TFC) at 149.02 ± 57.47 mg QE/g DW, condensed tannins (TCT) at 2.34 ± 0.171 mg CE/g DW, and flavonols at 81.67 ± 0.497 mg DE/g DW. In vitro tests showed significant antioxidant potential, with a total antioxidant capacity (TAC) of 44.11 ± 6.328 mg AAE/g DW, DPPH radical scavenging activity (IC_50_ = 4.89 mg/mL), and reducing power assay (FRAP, IC_50_ = 0.525 mg/mL). The results indicated that the extract significantly reduced body weight gain, plasma insulin levels (0.30 ± 0.00 U(IU)/mL), glycemia (0.955 ± 0.068 g/L), total cholesterol (0.555 ± 0.177 g/L), and triglycerides (0.720 ± 0.141 g/L). Regarding oxidative stress markers, the extract decreased levels of malondialdehyde (MDA, 4567 ± 121 μmol/L), hydroperoxides (1304 ± 288 μmol/L), and carbonylated proteins (0.029 ± 0.020 μmol/L), while increasing levels of vitamin C (25.84 ± 3.00 mg AAE/L), Oxygen Radical Absorbance Capacity (ORAC, 6.043 ± 0.345 UA), and catalase activity (0.0052 ± 0.00008 μL/mL). These findings suggest that *P. persica* L. may alleviate impairments related to metabolic syndrome by improving metabolic profiles and reducing oxidative stress in rats fed a high-fructose diet, making it a potential dietary supplement for managing metabolic syndrome.

## 1. Introduction

The metabolic syndrome has been defined by a cluster of interconnected metabolic alterations. It poses a major public health concern due to its association with the emergence of type 2 diabetes, cardiovascular disease, and other complications [1]. In the face of this alarming situation, the exploration of preventive and therapeutic strategies from natural sources has garnered increasing interest [2]. Phytotherapy, the use of plant-derived compounds for therapeutic purposes, has gained increasing attention as a complementary approach for managing various health conditions, including metabolic syndrome [3]. Phytotherapy offers several advantages, such as potential synergistic effects from a combination of multiple bioactive compounds, fewer side effects than synthetic drugs, and accessibility for populations with limited access to conventional health care. *Prunus persica* L., commonly known as the peach tree, is a plant that has attracted particular attention for its potential effects on metabolic syndrome. It is a deciduous plant of the Rosaceae family that is endemic to China. It is extensively grown for its edible fruits, which are renowned for their delicious taste and nutritional value. However, in addition to their culinary applications, various parts of the peach tree, including its leaves, bark, and kernels, have also been recognized for their potential medicinal properties [4].

*P. persica* L., also referred to as the peach tree, is a hardwood species that originates from the area of Northwest China [5]. Numerous bioactive substances, including flavonols, quercetin 3-glucoside, kaempferol 3-glucoside, kaempferol 3-galactoside, and quercetin 3-galactoside, are abundant in the peach plant. In a prior work, “HPLC” analysis was used to identify and quantify these flavonol components in peach leaf extracts [6]. These compounds exhibit potent pharmacological activities and health benefits, including antioxidant, anti-inflammatory, anticancer, and hypoglycemic properties. Several recent studies have explored the potential biological activities and therapeutic applications of *P. persica* leaf extract. In 2021, studies by Mostafa et al. [7] were conducted on aqueous and ethanol extracts of Florida Prince Cultivar peach leaves using DPPH, ABTS and β-carotene methods. The results obtained were promising but still inferior to those of other studies. According to their research, the leaves have greater antioxidant effects than seeds, pulp, peels and fruits. Antioxidant activity results from the presence of flavonols [7]. In 2019, Prakash and Sagar [8] examined the α-amylase inhibitory activity of peach leaf water, methanol and acetone extracts. The results showed that the plant extracts were effective in inhibiting α-amylase, with the methanol extract being more effective than the other solvent extracts. This study offers a rationale for the traditional usage of this plant to treat a number of illnesses, including obesity and diabetes [8]. In 2020, to assess the cytotoxic potential of two peach cultivars, Florida Prince and Desert Red Expanse, studies were conducted on their leaves. The outcomes demonstrated that the ethanol extracts contained vitamins C and E, suggesting a possible contribution to cytotoxic activity [9]. A study conducted in 2015 sought to assess the hepatoprotective potential of the ethanolic extract of the leaves of *P. persica* L. using carbon tetrachloride (CCl4)-treated rats. The results indicated that the ethanolic leaf extract of *P. persica* L. possesses hepatoprotective activity, potentially attributed to the presence of flavonoids [10].

Studies have indicated that peach leaves, which are part of the Rosaceae family, have properties that can inhibit tumor growth, reduce inflammation, alleviate allergies, and act as antioxidants [11]. In 2011, Bhattacharjee et al. [12] assessed *P. persica*. leaf aqueous extracts’ anti-inflammatory properties, particularly in carrageenan-induced edema. Their study revealed that *P. persica*. exhibits protective effects against inflammatory disorders in laboratory animals, a phenomenon also observed by traditional practitioners [12]. As comprehensively reviewed by Haleema et al. [13], extracts from various parts of the *P. persica*. plant, including leaves, bark, and kernels, have demonstrated promising effects in preclinical studies.

To demonstrate the potential protective effect of *P. persica* leaf extract against metabolic syndrome and oxidative stress in a rat model fed a high-fructose diet, the design of this study has two primary goals. First, it revealed the rich composition of bioactive substances such polyphenols, flavonoids, flavonols, and condensed tannins in the organic acidic extracts from this plant, as well as their phytochemical profile and possible antioxidant activity. Additionally, a study using animals was carried out to assess the in vivo effectiveness of peach leaf extract in reducing the oxidative stress and metabolic disturbances that excessive fructose consumption in rats caused. Histological examinations were performed to assess the impact of peach leaf extract on the architectural integrity and morphology of vital organs, including the liver, kidney, pancreas, spleen and perirenal adipose tissue, which are often compromised in metabolic disorders. This comprehensive approach aimed to provide a multifaceted understanding of the therapeutic potential of *P. persica* leaf extract, bridging its phytochemical characterization with its physiological and biochemical effects in a relevant disease model. The current work has the potential to facilitate the development of targeted therapy interventions or nutraceuticals designed to manage metabolic syndrome and its associated consequences.

## 2. Results and Discussion

### 2.1. Phytochemical Screening

Table 1 shows the findings of the extracts’ phytochemical screening, which revealed a significant presence of alkaloids, tannins, flavonoids, and terpenoids, with the absence of saponins and coumarins. This finding was consistent with the results obtained by Fellah et al. [14] and Benmehdi et al. [15]. Table 2 shows the extraction yield percentages as well as the total phenolic, flavonol, flavonoid, and condensed tannin concentrations. The results of the phytochemical screening shown in Table 2 revealed a remarkable 53.8% yield of phenolic compounds from the organic acidic extract of peach leaves, which is in notable contrast to previous findings obtained using different extraction methods. For instance, the yields reported in this study are significantly greater than those obtained by Shukla and Kant [16] for *Prunus persica* seed, who achieved yields of 2.8% for petroleum ether, 2.1% for ethyl acetate, 1.6% for ethanol, and 0.4% for chloroform extractions. These results also exceeded the results of Asnaashari et al. [17] on the same Rosaceae plant, who reported yields of approximately 15.43% and 11.87%, respectively, for the same solvent extractions. Furthermore, Dhingra et al. [18] and Kant et al. [19] reported corresponding yields of 9.75% for aqueous extracts. These comparisons underscore the significant enhancement in phenolic content achieved through the organic acid extraction method employed in this study.

Table 2 shows that the organic acid extract of *P. persica* leaves exhibited a fairly high total phenolic content of 273.36 mg GAE/g DW. These findings are superior to those published by Asnaashari et al. [17], who reported 108.64 mg GAE/g DW in the methanolic extract of *Rubus fructicosus* leaf, which is part of the Rosaceae family. Additionally, the total phenolic content obtained herein is greater than that reported by Kazan et al. [20] (67.85 ± 0.96 mg GAE/g DW) and by Maatallah et al. [21], who quantified total polyphenols in different mature and immature peach leaf varieties. Their concentrations in mature leaves varied from 25.34 to 35.64 mg GAE/g DW, whereas immature leaves ranged from 32.26 to 45.93 mg GAE/g DW.

The extract’s flavonoid content was 149.02 mg QE/g DW, which is higher than the values reported by Maatallah et al. [21], who obtained values that varied from 8.09 to 7.78 mg/g DW and 11.26 to 8.69 mg/g DW for different mature and immature peach leaf varieties. The condensed tannin content of 2.34 mg CE/g DW obtained in this study was significantly lower than that in previous reports. For instance, those of Song et al. [22] were conducted on the same genus as our plant, and were 73.95 ± 0.9 mg/g DW. These results are significantly greater than our findings.

The flavonol content of 81.67 mg (QE)/g DW obtained in this study is greater than the concentration of flavonols detected in the aqueous extract from *Prunus padus* (PPL) leaves, which was reported by Telichowska et al. [23] to be 3.85 ± 0.08 mg/100 g DW. In comparison, the aqueous leaf extract from *Prunus serotina* (PSL) exhibited a lower flavonol content, with the highest concentration being catechins at 1.01 ± 0.01 mg/100 g DW, Based on to the same study. The PPL leaves were found to contain a more diverse profile of flavonols, including rutin, quercetin, naringenin, and a prominent catechin at 2.07 ± 0.02 mg/100 g DW [23].

Table 2 depicts the antioxidant activity levels. The TAC levels (total antioxidant capacity) of the *P. persica* leaf organic acid extract was 44.11 mg AAE/g DW. Using the phosphomolybdenum method, Mokrani et al. [6] reported much higher TAC values between 185.6 and 318.2 mg AAE/g DW for acetone/water extracts of Algerian peach leaves. Regarding the DPPH assay, Table 2 demonstrates that a lower IC_50_ suggests more antioxidant activity. *P. persica* leaves organic acid extract exhibited promising free radical scavenging activity, with a value of IC_50_ at 4.89 mg/mL in the DPPH assay. The value was comparable to the value obtained of the synthetic antioxidant butylated hydroxytoluene (BHT), which showed an IC_50_ of 0.52 mg/mL. These findings are higher than the values reported in previous studies. Christabel et al. [24] and Dhingra et al. [18] reported that the ethyl acetate fraction of peach leaves extracts displayed a stronger inhibitory activity. The IC_50_ values were 0.29 mg/mL and 0.184 mg/mL, respectively. In contrast, Maatallah et al. [21] found that different extracts of peach leaves at different maturity stages had usually lower IC_50_ values against DPPH ranging from 0.09 to 0.20 mg/mL.

Our results confirm the significant free radical scavenging ability of peach leaf extract, which compares favorably with established antioxidant standards. The IC_50_ value of 4.89 mg/mL found in the current study is slightly more than that reported in prior investigations. This chemical has potential antioxidant activity and is equivalent to the results reported from other peach leaves extracts using the DPPH method. This action might be related to the extract’s phenolic components and other antioxidant constituents in the extract. According to the reducing power assay, the extract exhibited an IC_50_ value of 0.525 mg/mL, indicating its ability to reduce ferric ions and suggesting the presence of electron-donating antioxidants. This value was comparable to the value obtained of the synthetic antioxidant butylated hydroxytoluene (BHT), which showed an IC_50_ of 0.091 mg/mL.

The higher phenolic content and antioxidant capacities reported in the current study might be attributable to the specific organic acidic extraction conditions employed. The addition of organic acids such as acetic or citric acid to the extraction solvent has been shown to enhance the solubilization and recovery of phenolic compounds, particularly those bound to plant cell walls or present in glycosidic forms [6,25,26]. The acidic environment disrupts intermolecular interactions and hydrolyses glycosidic bonds, facilitating the release of phenolic aglycones and increasing their extractability. Variations in reported antioxidant capacities across studies may also arise from factors such as extraction solvents, plant cultivars/origins, and quantification assay methods. Nonetheless, the promising antioxidant properties observed, coupled with the phenolic compounds and flavonoids identified in the phytochemical screening, highlight the potential of *Prunus persica* L. leaf extract as a rich source of natural antioxidants.

### 2.2. Animal Experimentation

#### 2.2.1. Acute Toxicity Test

According to the results of the acute toxicity study per OECD guideline 423, *P. persica* leaf extract did not cause mortality in rats administered with various doses (25 mg/kg, 50 mg/kg, or 100 mg/kg) administered orally. Furthermore, no significant changes or physical signs of toxicity or mortality were observed in the behavior of the animals in any of the groups, even after 2 weeks. Therefore, it can be considered relatively safe.

#### 2.2.2. Oral Glucose and Insulin Tolerance Test (OGTT, ITT)

**Figure 1:**
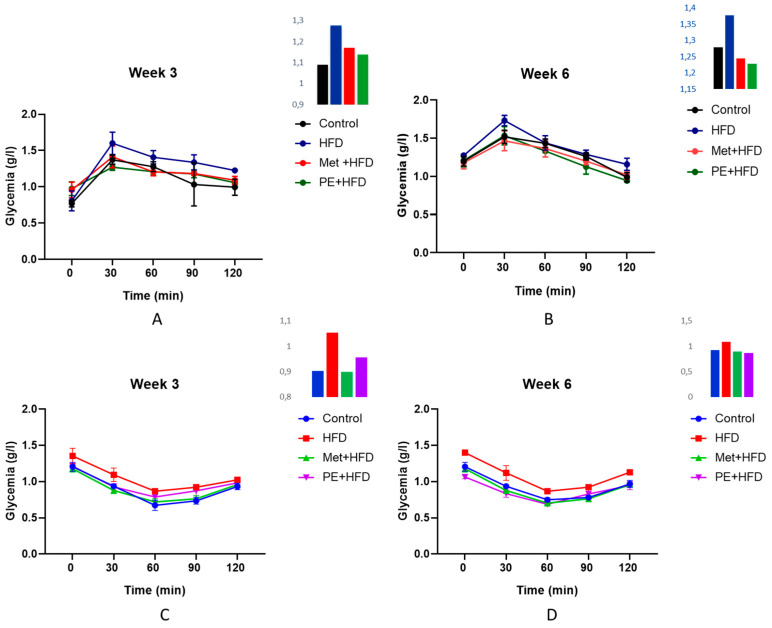
OGTTs (**A**,**B**) and ITTs (**C**,**D**) of control, fructose (HFD), metformin (Met + HFD) and fructose-treated *Prunus persica* extract (PE + HFD) rats. The graph offset is the AUC for the OGTT and ITT.

The oral glucose tolerance tests (OGTTs) and insulin tolerance tests (ITTs) conducted at weeks 3 and 6, as demonstrated in Figure 1, provide valuable insights into the progressive impact of the dietary interventions and treatments on insulin sensitivity and glucose homeostasis.

During the OGTTs, the high-fructose diet (HFD) group exhibited significantly elevated blood glucose levels at both time points (Figure 1A,B), indicating rapid onset and worsening of impaired glucose tolerance and insulin resistance induced by excessive fructose consumption. These findings align with previous studies demonstrating the detrimental effects of high-fructose diets on glucose metabolism and insulin action [27,28].

Notably, both the metformin (Met + HFD) and *P. persica* extract (PE + HFD) treatment groups demonstrated lower blood glucose excursions than did the HFD group at week 3 (Figure 1A), suggesting early protective effects against HFD-induced glucose dysregulation. By week 6 (Figure 1B), the therapeutic effects became more pronounced, with these groups approaching or even slightly outperforming the control group in glucose handling during the OGTT.

The ITTs further corroborated these findings. At week 3 (Figure 1C), the HFD group displayed markedly slower and less pronounced decreases in blood glucose levels following insulin administration, which is indicative of insulin resistance development. In contrast, the Met + HFD and PE + HFD groups had higher insulin sensitivity than the HFD group, however insulin sensitivity was not completely restored to control levels. By week 6 (Figure 1D), the interventions had more pronounced effects, with both treatment groups demonstrating further improvements in their ability to lower blood glucose levels in response to insulin, approaching the control group’s response.

#### 2.2.3. Body Weight

The high-fructose diet (HFD) group had considerably higher end body weights and weight gain than the control group on a regular diet. The increase in weight per week was consistent across all experimental groups, with the exception of the HFD rats, whose weight showed a slight increase from week 3 until the conclusion of the experiment (Table 3). This finding is consistent with previous studies reporting increased body weight and adiposity with high-fructose (30% *w*/*w*) feeding in rodents [29,30].

Table 3 shows the growth performance of each experimental group. The results indicated that the body weight diminished in the control, the metformin-treated (Met + HFD), and the *P. persica*-treated (PE + HFD) groups compared to the fructose group (HFD). Remarkably, the control group exhibited the most potent inhibition of body weight gain at 73.50 ± 15.29, followed by the PE + HFD group at 87.00 ± 9.42 and then the Met + HFD group at 95.00 ± 19.06. These values were considerably lower than those reported within the HFD group. These findings are consistent with prior research on other plant extracts from the *Prunus* genus that have shown antiobesity effects. For example, *Prunus mume* extract lowered body weight growth and ameliorated metabolic disturbances on obese diet-induced mice [31]. Similarly, an extract from *P. domestica* leaves decreased body weight and fat accumulation in obese rats [32].

#### 2.2.4. Insulinemia, Total Cholesterol, Triglycerides, Glycemia

This study looked at the possible preventive impact of peach leaf extract in mitigating metabolic syndrome generated in rats by a high-fructose diet, with metformin serving as a positive control treatment for comparison. Table 4 displays the results. As depicted in Table 4, the high-fructose diet (HFD) group displayed characteristic features of metabolic syndrome, including insulin resistance (elevated plasma insulin: 1.92 ± 1.65 U (IU)/mL), impaired glycemia (higher glycemia value: 1.462 ± 0.062 g/L), and dyslipidemia (increased triglycerides: 1.395 ± 0.035 g/L and total cholesterol: 0.745 ± 0.106 g/L), compared with the control group fed a standard diet. This finding aligns with previous studies reporting metabolic disturbances similar to those of high-fructose feeding in rodents. Pektaş et al. [31] explored how a high-fructose diet affected metabolic syndrome in rats. They found that a high-fructose diet caused considerable increases in body weight (g) (male: 415 ± 6.5; female: 260 ± 1.8), insulin resistance (ng/mL) (male: 4.38 ± 0.52; female: 4.97 ± 7.8), impaired glucose tolerance (mg/dL) (male: 129.4 ± 19.1; female: 113.2 ± 7.8), and dyslipidemia (mg/dL) (triglycerides: male: 132.8 ± 8.5; female: 205.1 ± 27.2, total cholesterol: male: 42.1 ± 1.4; female: 54.4 ± 5.3) compared to those in the control group.

Remarkably, supplementation with *P. persica* leaf extract (in the PE + HFD group) effectively alleviated most of these metabolic derangements. It reduced insulin resistance, lowering plasma insulin to 0.30 ± 0.00 U (IU)/mL, comparable to that in the metformin-treated group (Met + HFD: 0.360 ± 0.084 U (IU)/mL). Both treatments improved glycemic control, with the PE + HFD (0.955 ± 0.068 g/L) and Met + HFD (0.940 ± 0.181 g/L) groups exhibiting normal glycemia levels. Regarding the lipid profile, PE + HFD supplementation had promising hypolipidemic effects, significantly decreasing triglyceride levels (0.720 ± 0.141 g/L), similar to metformin (0.780 ± 0.156 g/L). Compared with the HFD, the plant extract also decreased the total cholesterol level (0.555 ± 0.177 g/L), although the difference was not statistically significant. Nonetheless, the control group exhibited the most potent inhibition of all parameters. The well-documented insulin-sensitizing and glucose-lowering effects of the pharmaceutical drug metformin have been reported Giannarelli et al. [33]. However, the comparable potential protective effects of *P. persica* leaf extract in mitigating insulin resistance and hyperglycemia are novel findings, corroborating previous research on other medicinal plant extracts. Eddouks et al. [34] present a review that emphasizes the numerous plants that have been shown to improve insulin sensitivity associated with diabetes.

#### 2.2.5. Determination of Oxidant/Antioxidant Status Parameters

As shown in Table 5, vitamin C levels were no significant differences between the groups. However, the PE + HFD group showed a trend toward higher levels (25.84 ± 3.00 mg AAE/L plasma) than did the HFD group (22.68 ± 7.10 mg AAE/L plasma), indicating potential antioxidant effects. Significant variations were observed in the total antioxidant capacity (ORAC) assay among the experimental groups. The HFD group displayed a markedly reduced ORAC value (1.985 ± 0.952 UI) compared to that of the control group (5.064 ± 0.134 UI), indicating a substantial decrease in antioxidant capacity induced by the high-fructose diet. Remarkably, the PE + HFD group exhibited the highest ORAC value (6.043 ± 0.345 UI) among all groups, surpassing even the ORAC value of the Met + HFD (5.246 ± 0.646 UI) group, suggesting superior antioxidant capacity in reducing oxidative damage caused by the high-fructose diet.

The catalase activity assay results revealed subtle differences among the experimental groups. The HFD group exhibited the lowest catalase activity (0.0037 ± 0.0001 μmol/L) compared to that of the control group (0.0051 ± 0.0002 μmol/L), indicating a diminished ability to detoxify hydrogen peroxide (H_2_O_2_) and counteract oxidative stress. In contrast, both the Met + HFD and PE + HFD groups showed restored catalase activity, with values of 0.0052 ± 0.00007 μmol/L and 0.0052 ± 0.00008 μmol/L, respectively; this suggests the effectiveness of *P. persica* leaf extract in restoring catalase activity and mitigating oxidative stress. Interestingly, both the Met + HFD and PE + HFD groups displayed lower catalase activity than the HFD group, which could be attributed to a reduced oxidative burden.

In comparison to the control group (3199 ± 509 μmol/L), the HFD group exhibited markedly elevated levels of malondialdehyde (MDA) (9471 ± 2125 μmol/L), a biomarker of oxidative stress and lipid peroxidation. This finding is consistent with previous studies reporting increased oxidative stress with high-fructose (30% *w*/*w*) feeding in rodents [29,35]. Interestingly, compared with those in the HFD group, the MDA levels in the control, Met + HFD, and PE + HFD treatment groups were significantly attenuated, suggesting that these treatments have antioxidant and antilipoperoxidative effects.

Notably, for the hydroperoxide levels, the control group and the PE + HFD group had the lowest values (1389 ± 460 μmol/L; 1304 ± 288 μmol/L), followed by the Met + HFD group (1533 ± 420 μmol/L) and the HFD group (2207 ± 413 μmol/L). No statistically significant differences were observed for carbonylated protein levels, as shown in Table 5, although the levels in the control (0.031 ± 0.020 μmol/L), Met + HFD (0.038 ± 0.016 μmol/L) and PE + HFD (0.029 ± 0.020 μmol/L) groups tended to decrease compared with those in the HFD group (0.067 ± 0.032 μmol/L). These findings corroborate previous studies that reported the potent antioxidant activity of *P. persica* extracts and the ability to modulate the activities of antioxidant enzymes, such as catalase. These antioxidant properties have also been observed in other species from the Rosaceae family, such as *Malva verticillata* [31] and *Prunus avium* [36]. This ability of *Prunus persica* L. to mitigate certain oxidative stress indicators caused by a high-fructose diet could be attributed as a result of its rich phytochemical composition, which includes polyphenols, flavonoids, and anthocyanins with well-documented antioxidant properties. These bioactive compounds are known to possess potent free radical scavenging, antioxidant, and enzyme-modulating properties [30,37,38].

Finally, this study provides insights into the antioxidant mechanisms potentially contributing to the potential protective effects of *P. persica* against metabolic syndrome. The results demonstrated the ability of *P. persica* leaf extract to restore antioxidant capacity, modulate antioxidant enzyme activities, and mitigate markers of oxidative stress and lipid peroxidation, with effects comparable or superior to those of the pharmaceutical drug metformin. These findings are supported by previous studies on the antioxidant properties of related species within the Rosaceae family, highlighting the potential protective effect of this plant extract in alleviating oxidative stress-related diseases, such as metabolic syndrome.

#### 2.2.6. Organ Weight

The weight of each organ for all the groups is represented in Table 6. Consistent with previous findings, the high-fructose diet (HFD) group exhibited significantly greater liver weight than the control group, which is indicative of hepatic dysfunction induced by excessive fructose consumption. Numerous studies have reported nonalcoholic fatty liver disease in rodents fed diets containing 30% or more fructose [29,30]. Remarkably, *P. persica* leaf extract supplementation (PE + HFD) significantly mitigated this effect, with liver weights comparable to those of the control group, suggesting that *P. persica* leaf extract has hepatoprotective effects against fructose-induced steatosis. Although not statistically significant, the PE + HFD group also showed a trend toward lower kidney weights than the HFD group, which could indicate potential renoprotective effects by ameliorating fructose-induced renal lipid accumulation and hypertrophy [39]. No significant differences were noted in the pancreas or spleen weights among the groups. Notably, the PE + HFD group exhibited significantly lower perirenal tissue weight than the HFD group, suggesting reduced accumulation of visceral adipose tissue. This finding aligns with the anti-obesogenic effects observed with *Prunus persica* L. supplementation in this study and corroborates previous research on the antiadipogenic potential of bioactive compounds from the *Prunus* genus [40]. These results line up with previously published studies that show the protective benefits of *Prunus* extracts against hepatic steatosis, renal complications, and excessive adipose tissue accumulation associated with metabolic disorders. *Prunus mume* extracts attenuated hepatic lipid accumulation, oxidative stress, and adipogenesis in high fat diet-fed mice [31]. The ability of *P. persica* to mitigate fructose-induced hepatic steatosis, visceral adiposity, and potentially renal complications could be attributed to its rich phytochemical profile, which includes polyphenols such as chlorogenic acid with well-established antioxidant, anti-inflammatory, and lipid-lowering properties [41,42].

#### 2.2.7. Histological Section Study

**Figure 2:**
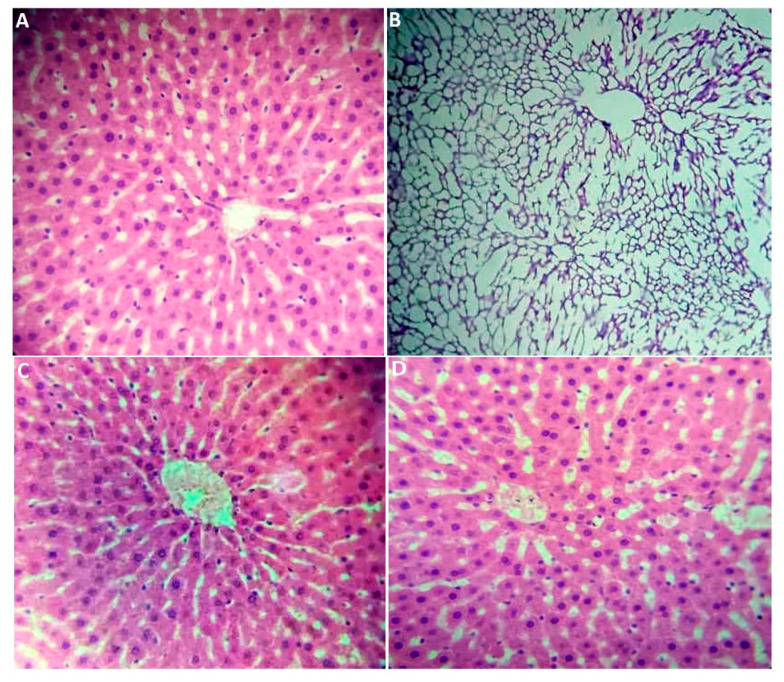
Histology of livers from the experimental groups (hematoxylin and eosin staining; (**A**,**C**,**D**) ×40 and (**B**) ×10). (**A**) Normal liver parenchyma in control rats, (**B**) alterations in the architecture of the hepatic parenchyma in HFD rats, (**C**) normal liver parenchyma in Met + HFD rats, and (**D**) normal liver parenchyma in PE + HFD rats.

The histological study involved the microscopic examination of tissue samples to investigate their cellular structure and organization. It has provided valuable insights into physiological processes, structural abnormalities, and potential disease states within the examined tissues, contributing to a deeper understanding of biological mechanisms and informing clinical diagnoses and therapeutic approaches [43]. In fact, Figure 2 shows that microscopic observation of the liver revealed significant alterations in the architecture of the hepatic parenchyma. Except for the hepatic capsule, the connective tissue separating the hepatic lobules contained only fibrous elements, while vascular elements such as branches of the hepatic artery, portal vein, and lymphatic vessels were absent. Additionally, Remak’s strands, which are typically composed of two rows of hepatocytes, were also missing. The hepatic lobule is distorted, appearing empty. Macroscopic observation of the liver from the HFD group revealed hepatomegaly, characterized by a large increase in the volume of the liver. After staining, examination of the slides revealed samples with a clear appearance. Notably, the other groups exhibit a completely normal parenchyma, with the absent elements in the HFD group being present in the control, PE + HFD, and Met + HFD groups. These groups presented a normal and well-organized hepatic parenchyma. The architecture of the hepatic lobule was maintained, with the presence of peri-lobular connective tissue containing various vascular elements at its periphery. In the center of the lobule, the central vein was visible, from which Remak’s strands extend, separated by sinusoidal spaces.

**Figure 3:**
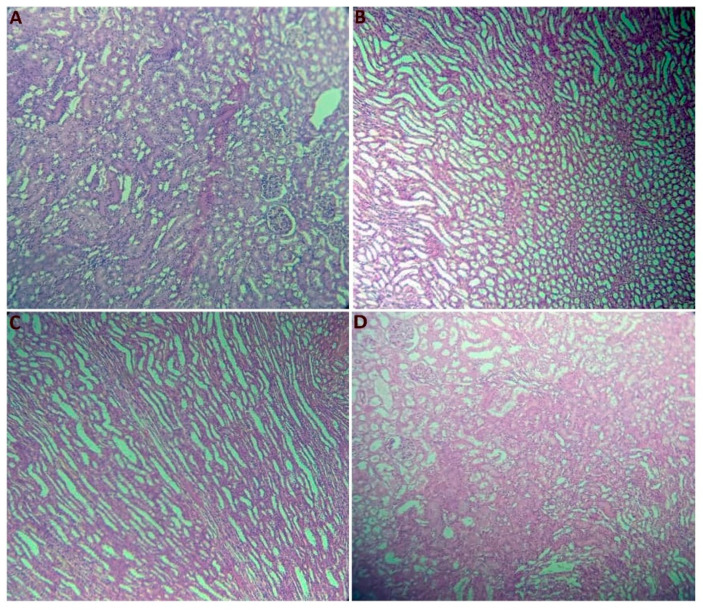
Histology of kidneys from the experimental groups (hematoxylin and eosin staining; ×10). (**A**) Normal renal parenchyma in control rats, (**B**) normal renal parenchyma in HFD rats, (**C**) normal renal parenchyma in Met + HFD rats, and (**D**) normal renal parenchyma in PE + HFD rats.

Figure 3 shows that the renal parenchyma exhibited well-preserved and normal architecture across all four experimental groups. Both the cortical and medullary zones were clearly discernible, showcasing a rich array of tubular and vascular elements. Within the cortical zone, characterized by a darker hue, prominent glomeruli were evident. Conversely, the medullary zone, which had a lighter appearance, featured the delicate and robust segments of the loop of Henle and a portion of the collecting tubule. These observations remained consistent across all of the experimental conditions.

**Figure 4:**
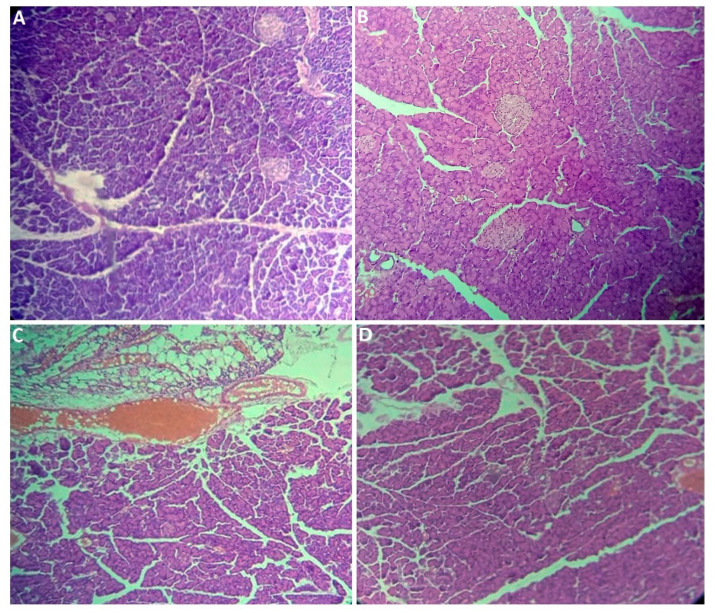
Histology of pancreata from the experimental groups (hematoxylin and eosin staining; ×10). (**A**) Normal pancreatic parenchyma in control rats, (**B**) normal pancreatic parenchyma in HFD rats, (**C**) normal pancreatic parenchyma in Met + HFD rats, and (**D**) normal pancreatic parenchyma in PE + HFD rats.

**Figure 5:**
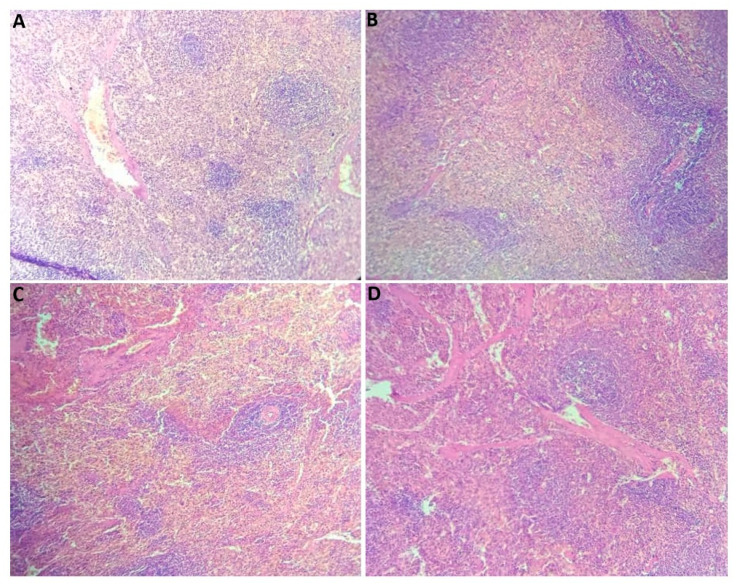
Histology of spleens from the experimental groups (hematoxylin and eosin staining; ×10). (**A**) Normal splenic parenchyma in control rats, (**B**) normal splenic parenchyma in HFD rats, (**C**) normal splenic parenchyma in Met + HFD rats, and (**D**) normal splenic parenchyma in PE + HFD rats.

**Figure 6:**
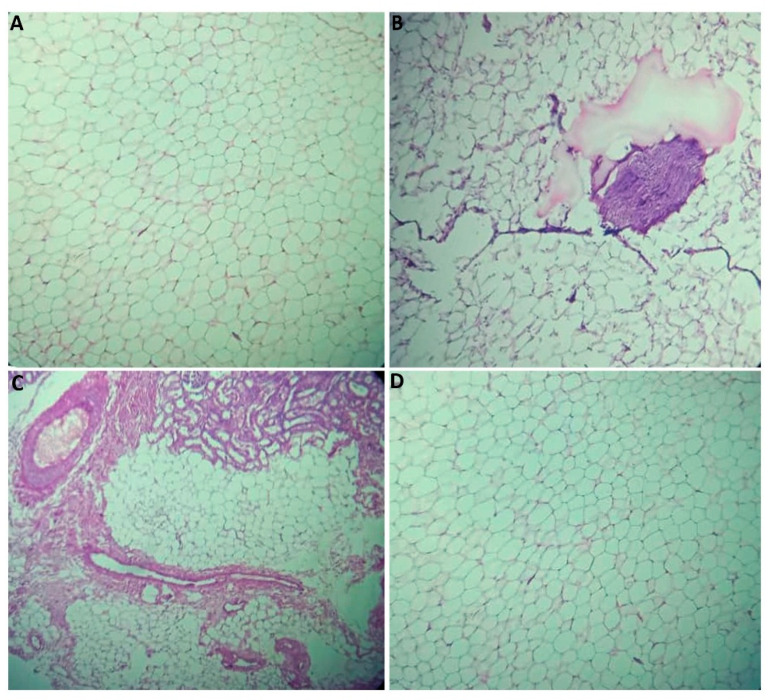
Histology of the perirenal tissue from the experimental groups (hematoxylin and eosin staining; ×10). (**A**) Normal perirenal tissue parenchyma in control rats, (**B**) infiltration of lymphocytes and fibrous formations in HFD rats, (**C**) normal perirenal tissue parenchyma in Met + HFD rats, and (**D**) normal perirenal tissue parenchyma in PE + HFD rats.

The pancreatic parenchyma shown in Figure 4 exhibited well-organized pancreatic lobules, each containing acini interspersed with clusters of Langerhans islets. The histological architecture of the splenic parenchyma represented in Figure 5 was well preserved across all of the experimental groups. It comprises two distinct splenic pulp regions: the arterial pulp, characterized by Malpighian corpuscles, and the venous pulp, consisting of Billroth cords and sinusoidal capillaries. These features were consistently observed across all research groups. The perirenal tissue parenchyma (Figure 6) showed strong preservation in the Control, Met + HFD, and PE + HFD groups, with adipocytes standing out. These adipocytes are identifiable by their characteristic hexagonal shape and eccentrically positioned nucleus, which are indicative of lipid vacuoles. Conversely, in the HFD group, significant infiltration of lymphocytes and fibrous formations was observed within much of the adipose tissue. This infiltration imparts a fibrotic appearance, disrupting the architectural integrity of the perirenal adipose tissue.

## 3. Materials and Methods

This study was approved by the Scientific Research Ethics Committee at Tlemcen University, Tlemcen, Algeria (Reference No: KSU-SE-22-41), and was carried out in accordance with the approved guidelines. All animal experiments followed the ARRIVE guidelines and were carried out in accordance with the National Institutes of Health guide for the care and use of Laboratory animals (NIH Publications No. 8023, revised 1978).

### 3.1. Plant Material

*P. persica* leaves were collected in September 2022 from the northwest Algerian province of Tlemcen. The leaves that were gathered were identified at the Laboratory of Botany (Abou Bakr Belkaid University of Tlemcen). The leaves were chopped into little bits after being carefully cleaned under running water and allowed to dry at room temperature away from the sun. The leaf pieces were powdered using a grinder.

### 3.2. Phytochemical Screening

Phytochemical screening was conducted to detect the presence or absence of various families of secondary metabolites. For this purpose, various compound groups’ characterization tests were run on the extracted materials. Phytochemical tests were conducted on the acidic organic extract according to the methods outlined previously [44].

### 3.3. Preparation of Extracts

An organic acidic extract of *P. persica* leaves (PE) was prepared following this procedure: 5 g of the powdered leaves were added to 75 mL of a solvent mixture comprising acetonitrile, water, and formic acid in a 49.5:49.5:1 ratio. This mixture was then subjected to ultrasonication for one hour at a frequency of 130 Hz. After ultrasonication, 75 mL of a 37% hydrochloric acid (HCl) solution was added for acidification. The obtained acidified mixture was subsequently heated in a water bath for an hour at 100 °C. Subsequently, the extract was refrigerated and allowed to macerate for 72 h. Then, the extract was filtered to remove any solid particles. A rotary evaporator was used to evaporate the filtrate, yielding the concentrated organic acidic extract of *P. persica* leaves.

#### 3.3.1. Determination of Total Phenolic Content

The total phenolic content in all extracts was assessed using the method published by Ainsworth & Gillespie [45]. Put simply, 200 μL of the extract was thoroughly combined with 1 mL of Folin–Ciocalteu reagent and diluted 10 times. After 5 min, 0.8 mL of 7.5% Na_2_CO_3_ solution was added to the mixture, which was left to incubate in the dark for 30 min at 25 °C. A spectrophotometer set to 765 nm was used to determine the absorbance. The total phenolic content was calculated as milligrammes of gallic acid equivalents (GAE) per gramme of dry weight. The samples were processed in triplicate.acid equivalents (GAE) per g of dry weight (DW). The samples were processed in triplicate.

#### 3.3.2. Determination of Flavonoid Content

According to protocols from Belyagoubi-Benhammou et al. [46], the flavonoids were quantified as follows: 500 μL of plant extract was added to 1.5 mL of distilled water, followed by 150 μL of 5% NaNO_2_ solution. After 5 min of incubation at 25 °C, 150 μL of 10% AlCl_3_ was added. Following another 6 min of incubation at 25 °C, 0.5 mL of 1 M NaOH was added to the resulting mixture. The absorbance was then measured at 510 nm. The flavonoid concentration was calculated from a quercetin standard curve and expressed as mg QE/g DW. Each sample was analyzed in triplicate.

#### 3.3.3. Determination of Condensed Tannins

The examination of condensed tannins was carried out using the methods described by Benmeliani-Yousfi et al. [47]. A solution was prepared by combining 3 mL of 4% vanillin in methanol, 1.5 mL of concentrated hydrochloric acid, and 400 μL of a suitably diluted sample. This mixture was incubated at room temperature for 15 min to allow the reaction to occur. After the incubation period, the absorbance of the solution was measured at a wavelength of 500 nm using a spectrophotometer. The total amount of condensed tannins present in the original sample was quantified by expressing the results in terms of milligrams of catechin equivalent per gram of dry weight (mg CE/g DW). The analysis was performed in triplicate for each sample to ensure reproducibility.

#### 3.3.4. Determination of Flavonols

The total flavonol content in the plant extracts was determined using the method described by Kumaran and Karunakaran [48]. To a 1 mL aliquot of the sample, 1 mL of 2% aluminum chloride (AlCl_3_) in ethanol and 3 mL of a 50 g/L sodium acetate solution were added. The mixture was incubated at 25 °C for 2.5 h to allow the reaction to proceed. After the incubation period, the absorbance of the solution was measured at 440 nm using a spectrophotometer. The total flavonol content was quantified by expressing the results in terms of milligrams of quercetin equivalent per gram of dry weight (mg QE/g DW). This analysis was performed in triplicate for each sample to ensure reproducibility.

### 3.4. Antioxidant Activity

#### 3.4.1. DPPH Radical Scavenging Activity Assay

The DPPH (2,2-diphenyl-1-picrylhydrazyl) radical scavenging activity assay was performed as follows. For the plant extracts, 50 μL aliquots of various concentrations were added to 1950 μL of a methanolic DPPH solution (0.025 g/L). The mixture was vortexed thoroughly and incubated in the dark at room temperature for 30 min to allow the reaction to occur. After the incubation period, the absorbance of the solution was measured at 515 nm using a spectrophotometer. The radical scavenging activity was calculated as the percentage of discoloration of the DPPH solution using the following equation:

DPPH^•^ scavenging-radical (%) = [(A0 − As)/A0] × 100,

where A0 is the absorbance of the control reaction and As is the absorbance of the sample solution containing the test compound.

BHT was used as a positive control. The IC_50_ of the extract was calculated from the percentage of inhibition plotted against the extract concentration. Measurements were made in triplicate [49].

#### 3.4.2. Reducing Power

According to the method described by Vijayalakshmi & Ruckmani [50], various amounts of the extracts were dissolved in distilled water and mixed with 2.5 mL of 0.2 M phosphate buffer (pH 6.6) and 2.5 mL of 1% potassium ferricyanide solution. The resulting mixture was incubated at 50 °C for 20 min. After the incubation period, 2.5 mL of 10% trichloroacetic acid was added to the mixture, which was then centrifuged for 10 min. Subsequently, 2.5 mL of the supernatant was mixed with 2.5 mL of distilled water and 0.5 mL of a freshly prepared 0.1% ferric chloride (FeCl_3_) solution. The absorbance of the final solution was measured at 700 nm using a spectrophotometer. Butylated hydroxytoluene (BHT) was used as a positive control. In this assay, a higher absorbance value corresponds to a higher reducing power. The analysis was performed in triplicate for each concentration of the extracts to ensure reproducibility.

#### 3.4.3. Total Antioxidant Capacity

According to the methods described by Benmahieddine et al. [51], various amounts of the extracts were dissolved in distilled water and mixed with 2.5 mL of 0.6 M sulfuric acid, 2.5 mL of 28 mM sodium phosphate, and 2.5 mL of 4 mM ammonium molybdate. The resulting mixture was incubated at 95 °C for 90 min to allow the reaction to proceed. After cooling to room temperature, the absorbance of the solution was measured at 695 nm using a spectrophotometer. Ascorbic acid was used as a positive control. In this assay, a higher absorbance value corresponds to a higher total antioxidant capacity. The analysis was performed in triplicate for each sample to ensure reproducibility.

### 3.5. Experimental Animals

Male *Wistar* rats (200–250 g) were kept under conventional conditions in the laboratory (25 ± 2 °C, 50 ± 15% relative humidity, and a natural dark/light phase). There was unlimited access to food and water. An electronic balance was used to measure the body weight three times each week.

#### 3.5.1. Acute Toxicity Test

To mitigate any potential risks of toxicity during biological testing, it was imperative to conduct toxicity assays Following the guidelines of the Organisation for Economic Cooperation and Development (OECD) No. 423. For this aim, three different dosages of the extract were tested on a group of nine rats, with three animals per dose and equal body weights. The rats received single oral doses of 25 mg/kg, 50 mg/kg, or 100 mg/kg on the first day of treatment. After administration, the animals were intensively monitored individually for the first 30 min, followed by regular observations every 4 h for the first 24 h, with specific emphasis on the first 4 h. The rats were then monitored daily for 14 days, with any symptoms of discomfort, ill effects, or mortality recorded. The rats were maintained in a controlled setting at room temperature, three per cage. During the observation period, the rats had free access to water and typical rodent food.

Careful monitoring and adherence to the OECD guidelines ensured a systematic approach for assessing the potential toxicity of the extracts, allowing for the identification of any adverse effects and determining safe dose ranges for further biological testing.

#### 3.5.2. Preparation of a High-Fructose Diet

In this research, a rat model of metabolic syndrome was developed using D-Fructose with a purity of more than 99%. Every two days, 30 g of fructose was diluted in 100 mL of tap water to make a final concentration of 30% fructose. To avoid fermentation, the water bottles were wrapped with aluminum foil. The rats were fed the high-fructose diet (HFD) ad libitum for 6 weeks in order to induce metabolic syndrome [52].

#### 3.5.3. Experimental Design

After one week of adjustment, 16 rats were distributed at random to four distinct experimental groups as follows (n = 4). The control group, rats were fed with a standard rodent diet. In the high-fructose diet (HFD) group, the rats were fed a standard diet + the 30% fructose solution in water (ad libitum). The rats in the metformin-treated group (Met + HFD) received metformin (50 mg/kg) + the 30% fructose solution, which was administered orally twice a day. *P. persica*-treated rats (PE + HFD) were fed a standard diet + the 30% fructose solution + 2 mL/d of *P. persica* extract (oral administration). For a 6-week experimental period.

#### 3.5.4. Oral Glucose Tolerance Test and Insulin Tolerance Test

Oral glucose tolerance test (OGTT) was carried out at 3 and 6 weeks into the study in the control rats, fructose-high diet control rats (HFD), fructose-high diet-fed rats treated with metformin (MET + HFD) and fructose-high diet-fed rats treated with *P. persica* extract (PE + HFD). Samples of blood were collected from the tail vein of fasted rats (5 h). The blood samples were collected at 0, 15, 30, 60, 90, and 120 min after the oral administration of 5 mL of glucose solution (3 g/kg). Glucose was measured using a glucometer (Vital-Chek^®^ MM1200 blood glucose meter). Insulin tolerance test (ITT) was conducted in the same groups at 3 and 6 weeks of age as mentioned above. After a 5 h fast, the rats received an intraperitoneal injection of 0.5 U/kg insulin. Blood samples were taken via the tail vein at 0, 15, 30, 60, 90, and 120 min. Glucose was tested using the same glucometer as mentioned above.

#### 3.5.5. Animal Euthanasia

After a 6-week experimental period and an overnight fast (12 h), the rats were then euthanized by an anesthetic overdose (intraperitoneal injection of ketamine at 100 mg/kg and gardenal tranquilizer at 40 mg/2 mL). Blood sampling was performed by cardiac puncture into heparinized hemolysis tubes. The heparinized blood was then centrifuged to recover the plasma (2200× *g* for 10 min at room temperature). The plasma tubes were stored in a freezer until analysis. The remaining pellet was washed with physiological saline, and then the erythrocytes were lysed by the addition of chilled distilled water. Centrifuged for 10 min at 4000 rpm to remove cellular debris. The lysate was then recovered and stored in a freezer until analysis. The liver, pancreas, perirenal tissue, kidneys, and spleen were collected, washed in a 9% NaCl solution, weighed and then fixed in formalin for histological sectioning.

### 3.6. Biochemical Study

Glucose, triglyceride, and total cholesterol serum levels were assessed using commercial kits (Sarl Sigma Prochima). Levels of serum insulin were measured using the ELISA test (enzyme-linked immunosorbent assay).

#### Determination of Oxidant/Antioxidant Status Parameters

Oxidative stress, defined as an imbalance between the body’s antioxidant defense mechanisms and the formation of reactive oxygen species (ROS), is a critical factor in a variety of clinical disorders. To evaluate the oxidant/antioxidant status in blood samples, several parameters are commonly assessed.

The vitamin C test determines the ascorbic acid levels, a powerful water-soluble antioxidant that scavenges free radicals and regenerates other antioxidants [53]. The ORAC assay (oxygen radical absorbance capacity) assesses a sample’s total antioxidant capacity by measuring its ability to neutralize peroxyl radicals [54].

The catalase test measures the activity of the catalase enzyme, which induces the dissolution of hydrogen peroxide, a toxic ROS, into water and oxygen. It was measured by spectrophotometric monitoring the rate of hydrogen peroxide (H_2_O_2_, 35 mM) decomposition at 240 nm by catalase enzyme (CAT100-1KT, Sigma-Aldrich) [55].

Additionally, the assessment of carbonylated proteins, which are proteins modified by oxidative stress, provides insights into oxidative damage to macromolecules [56]. The malondialdehyde (MDA) assay measures the concentration of MDA, a result of lipid peroxidation and a commonly used indicator of oxidative stress [57]. Complementary to the assessment of antioxidant status, the evaluation of hydroperoxide levels is essential [58]. They were estimated by specific methods using thiobarbituric acid or 2,4-dinitrophenyl hydrazine reaction, respectively (MAK085-1KT and MAK094-1KT, respectively, Sigma-Aldrich kit; St. Louis, MO, USA).

### 3.7. Histological Section Study

After the rats were sacrificed, the liver, kidneys, pancreas, spleen, and perirenal tissue were weighed, dissected, and fixed in 10% formalin. The study of histological sections was conducted at the Histology–Embryology Laboratory, Faculty of Medicine Dr. Benzerdjeb Benaouda, University of Tlemcen. The tissue samples were dehydrated, cleared, and inserted into paraffin blocks using conventional histological procedures. Thin, 3–5 micron-thick sections were cut from the blocks using a microtome and placed on glass slides. The cut sections were then deparaffinized in xylene/toluene, rehydrated with decreasing ethanol, stained with hematoxylin and eosin (H&E), followed by dehydration. The stained sections were mounted with coverslips and examined microscopically at 10× and 40× with a Leica microscope. for observation and interpretation of cellular structures, tissue types, and pathologies against histological standards [43].

### 3.8. Statistical Analysis

The findings were evaluated using one-way ANOVA, *p* values < 0.05 were considered significant. The studies were conducted by using Graph Pad Prism version 8.

## 4. Conclusions

This research highlights the possibility of employing by-products such as leaves from the *Prunus persica* L. tree for therapeutic purposes, namely in addressing metabolic imbalances and oxidative stress produced by a high-fructose diet. Due to its richness in total phenolics, flavonoids, and flavonols, the extract demonstrated an interesting total antioxidant capacity, antiradical activity toward DPPH, and reducing power. Additionally, the extract showed a decrease in insulin resistance and revealed hypolipidemic effects by reducing levels of plasma insulin, glycemia, triglycerides, and total cholesterol. Furthermore, it enhanced the activation of antioxidant indicators (vitamin, ORAC, and catalase), while inhibiting oxidative stress indicators (MDA, hydroperoxides, and carbonylated proteins). The extract’s success in lowering metabolic and histological abnormalities can be linked to its high concentration of bioactive components. These discoveries create opportunities for specific therapy strategies and the creation of nutraceuticals designed to control metabolic syndrome and its associated consequences.

## Figures and Tables

**Table 1:** Phytochemicals detected in extracts of *P. persica* leaves.

Compounds	Reactive	*P. persica* Extract
Alkaloids	Mayer	+++
	Wagner	+++
Tannins	FeCl_3_	++
Flavonoids	Shinoda test (Mg)	++
Coumarins	UV Fluorescence	-
Terpenoids	Slakowski Test	+
Saponins	Foam Test	-

(+++): Strongly present; (++): Moderately present; (+): Weakly present; (-): Negative test.

**Table 2:** Phytochemical contents and the antioxidant activity of *P. persica* extracts.

	Contents	IC_50_ (mg/mL)	
		DPPH Test	Reducing Power
**Yield** (%)	53.8		
**Total Phenolics** (mg GAE/g DW)	273.36 ± 1.929		
**Flavonoids** (mg QE/ g DW)	149.02 ± 57.478		
**Condensed Tannins** (mg CE/g DW)	2.34 ± 0.171		
**Flavonols** (mg QE/g DW)	81.67 ± 0.497		
**TAC** (AAE/g DW)	44.11 ± 6.328		
Extract		4.89 ± 0.010	0.525 ± 0.059
BHT		0.53 ± 0.040	0.091 ± 0.48

**GAE**: Gallic Acid Equivalent, **QE**: Quercetin Equivalent, **CE**: Catechin Equivalent, **DW**: Dry Weight, **TAC**: Total Antioxidant Capacity, **AAE**: Ascorbic Acid Equivalent, **BHT**: Butylated Hydroxytoluene, **IC_50_**: Half maximal Inhibitory Concentration, **DPPH**: 2,2-diphenyl-1-picrylhydrazyl.

**Table 3:** Growth performance of the experimental groups one way ANOVA.

	Control	HFD	Met + HFD	PE + HFD	DF	f	*p*-Value
Initial body weight (g)	207 ± 4.69 ^b^	214.25 ± 6.45 ^b^	231 ± 4.69 ^a^	234.50 ± 7.14 ^a^	3	15.29	0.000
Final body weight (g)	280.50 ± 11.82 ^b^	345.50 ± 12.15 ^a^	326 ± 22.50 ^a^	321.50 ± 6.45 ^a^	3	10.72	0.000
Body weight gain (g)	73.50 ± 15.29 ^b^	131.25 ± 15.24 ^a^	95.00 ± 19.06 ^b^	87.00 ± 9.42 ^b^	3	7.97	0.000

**Control**: Control rats, **HFD**: High fructose diet rats, **Met + HFD**: Metformin-treated fructose rats, **PE + HFD**: *P. persica*-treated fructose rats. The values are expressed as the means (SEMs) at 6 weeks. Different letters indicate significant differences.

**Table 4:** Plasma biochemical parameters in the experimental groups one way ANOVA.

	Control	HFD	Met + HFD	PE + HFD	DF	F	*p*-Value
**Total Cholesterol** (g/L)	0.525 ± 0.092 ^a^	0.745 ± 0.106 ^a^	0.595 ± 0.0636 ^a^	0.555 ± 0.177 ^a^	3	1.29	0.392
**Triglycerides** (g/L)	0.630 ± 0.042 ^b^	1.395 ± 0.035 ^a^	0.780 ± 0.156 ^b^	0.720 ± 0.141 ^b^	3	0.196	0.011
**Insulinemia** (U(IU)/mL)	1.32 ± 1.44 ^a^	1.92 ± 1.65 ^a^	0.360 ± 0.085 ^a^	0.30 ± 0.00 ^a^	3	1.02	0.471
**Glycemia** (g/L)	0.847 ± 0.091 ^b^	1.462 ± 0.062 ^a^	0.940 ± 0.182 ^b^	0.955 ± 0.068 ^b^	3	36.32	0.000

**Control**: Control rats, **HFD**: High-fructose diet rats, **Met + HFD**: Metformin-treated fructose rats, **PE + HFD**: *P. persica*-treated fructose rats. The values are expressed as the means (SEMs) at 6 weeks. Different letters indicate significant differences.

**Table 5:** Plasma and erythrocyte Oxidative stress markers in Experimental Groups.

	Control	HFD	Met + HFD	PE + HFD	*p*-Value
**Vit C** (mg AAE/L plasma)	22.980 ± 1.495 ^a^	22.68 ± 7.10 ^a^	23.22 ± 4.10 ^a^	25.84 ± 3.00 ^a^	0.478
**Catalase** (μmol/L)	0.0051 ± 0.0002 ^a^	0.0037 ± 0.0001 ^b^	0.0052 ± 0.00007 ^a^	0.0052 ± 0.00008 ^a^	0.000
**ORAC** (UI)	5.064 ± 0.134 ^a^	1.985 ± 0.952 ^b^	5.246 ± 0.646 ^a^	6.043 ± 0.345 ^a^	0.000
**MDA** (μmol/L)	3199 ± 509 ^b^	9471 ± 2125 ^a^	3574 ± 910 ^b^	4567 ± 1219 ^b^	0.000
**Hydroperoxides** (μmol/L)	1389 ± 460 ^b^	2207 ± 413 ^a^	1533 ± 420 ^b^	1304 ± 288 ^b^	0.044
**Carboniled-proteins** (μmol/L)	0.031 ± 0.020 ^b^	0.067 ± 0.032 ^a^	0.038 ± 0.016 ^a^	0.029 ± 0.020 ^a^	0.168

**Control**: Control rats, **HFD**: High-fructose diet rats, **Met + HFD**: Metformin-treated fructose rats, **PE + HFD**: *P. persica*-treated fructose rats. The values are expressed as the means (SEMs) at 6 weeks. Different letters indicate significant differences.

**Table 6:** Weights of the liver, kidney, pancreas, spleen, and perirenal tissue of the experimental groups.

	Control	HFD	Met + HFD	PE + HFD	*p*-Value
Liver	8.532 ± 1.193 ^ab^	10.043 ± 0.1895 ^a^	9.627 ± 1.245 ^ab^	7.716 ± 0.523 ^b^	0.016
Kidneys	1.043 ± 0.351 ^b^	2.1060 ± 0.0636 ^a^	1.988 ± 0.229 ^a^	1.7993 ± 0.0511 ^a^	0.000
Pancreas	0.943 ± 0.270 ^a^	0.984 ± 0.208 ^a^	0.890 ± 0.203 ^a^	0.927 ± 0.224 ^a^	0.949
Spleen	0.8540 ± 0.0998 ^a^	1.147 ± 0.359 ^a^	0.9748 ± 0.1658 ^a^	0.8453 ± 0.1804 ^a^	0.242
Peri-renal tissue	2.002 ± 1.124 ^ab^	3.638 ± 0.331 ^a^	2.071 ± 0.926 ^ab^	1.837 ± 0.893 ^b^	0.043

**Control**: Control rats, **HFD**: High fructose diet rats, **Met + HFD**: Metformin-treated fructose rats, **PE + HFD**: *P. persica*-treated fructose rats. The values are expressed as the means (SEMs) at 6 weeks. Different letters indicate significant differences.

## Data Availability

All materials in the manuscript are owned by the authors.
